# Efficacy in asthma of once-daily treatment with fluticasone furoate: a randomized, placebo-controlled trial

**DOI:** 10.1186/1465-9921-12-132

**Published:** 2011-10-06

**Authors:** Ashley Woodcock, Eric D Bateman, William W Busse, Jan Lötvall, Neil G Snowise, Richard Forth, Loretta Jacques, Brett Haumann, Eugene R Bleecker

**Affiliations:** 1 School of Translational Medicine, Manchester Academic Health Science Centre, University of Manchester, Manchester, UK; 2 Department of Medicine, University of Cape Town, Cape Town, South Africa; 3 Department of Medicine, University of Wisconsin, Madison, USA; 4 Krefting Research Centre, University of Gothenberg, Gothenberg, Sweden; 5 Respiratory Medicines Development Centre, GlaxoSmithKline, Uxbridge, UK; 6 Respiratory Medicines Development Center, Research Triangle Park, NC, USA; 7 Center for Genomics and Personalized Medicine, Wake Forest University Health Sciences Winston-Salem, NC, USA

**Keywords:** once-daily, ICS, asthma

## Abstract

**Background:**

Fluticasone furoate (FF) is a novel long-acting inhaled corticosteroid (ICS). This double-blind, placebo-controlled randomized study evaluated the efficacy and safety of FF 200 mcg or 400 mcg once daily, either in the morning or in the evening, and FF 200 mcg twice daily (morning and evening), for 8 weeks in patients with persistent asthma.

**Methods:**

Asthma patients maintained on ICS for ≥ 3 months with baseline morning forced expiratory volume in one second (FEV_1_) 50-80% of predicted normal value and FEV_1 _reversibility of ≥ 12% and ≥ 200 ml were eligible. The primary endpoint was mean change from baseline FEV_1 _at week 8 in pre-dose (morning or evening [depending on regimen], pre-rescue bronchodilator) FEV_1_.

**Results:**

A total of 545 patients received one of five FF treatment groups and 101 patients received placebo (intent-to-treat population). Each of the five FF treatment groups produced a statistically significant improvement in pre-dose FEV_1 _compared with placebo (p < 0.05). FF 400 mcg once daily in the evening and FF 200 mcg twice daily produced similar placebo-adjusted improvements in evening pre-dose FEV_1 _at week 8 (240 ml vs. 235 ml). FF 400 mcg once daily in the morning, although effective, resulted in a smaller improvement in morning pre-dose FEV_1 _than FF 200 mcg twice daily at week 8 (315 ml vs. 202 ml). The incidence of oral candidiasis was low (0-4%) and UC excretion was comparable with placebo for all FF groups.

**Conclusions:**

FF at total daily doses of 200 mcg or 400 mcg was significantly more effective than placebo. FF 400 mcg once daily in the evening had similar efficacy to FF 200 mcg twice daily and all FF regimens had a safety tolerability profile generally similar to placebo. This indicates that inhaled FF is an effective and well tolerated once-daily treatment for mild-to-moderate asthma.

**Trial registration:**

NCT00398645

## Background

Despite the availability of effective preventative therapies, asthma remains a major global healthcare problem, placing a significant burden on healthcare systems, patients and their families [1,2]. According to the World Health Organization, approximately 15 million disability-adjusted life years are lost annually due to asthma and approximately 1 in every 250 deaths worldwide are attributable to the disease [3].

As the cornerstone of anti-inflammatory therapy for all severities of asthma, inhaled corticosteroids (ICS) provide a number of benefits including control of asthma symptoms, improvement in lung function, decrease in airway hyper-responsiveness [4], reductions in asthma exacerbations, and reduced asthma mortality [5,6]. As a reflection of this, the current Global Initiative for Asthma guidelines recommend an ICS as a first-line controller therapy for asthma patients of all ages, who are not controlled on an as-needed, rapid acting beta_2 _agonist [2].

Despite comprehensive guidelines, a significant proportion of patients continue to have asthma symptoms that remain uncontrolled [7,8]. Twenty-four hour coverage might be expected to provide greater asthma control; however, the complexity of asthma treatment regimens and consequent poor adherence to treatment have been cited as major contributing factors to the current poor level of global asthma control [2,9,10]. Once-daily treatments offer increased convenience, with the potential for improved adherence and asthma control [11]. Many of the commonly prescribed ICS therapies for asthma, including beclomethasone dipropionate, flunisolide, ciclesonide and fluticasone propionate, are indicated for twice-daily dosing; however, once-daily administration has been investigated in some ICS including budesonide [12-14], mometasone furoate [15], and ciclesonide [16]. These studies have indicated that once daily evening administration is at least as effective as once daily morning administration with respect to PEF [13], or results in greater FEV_1_/FVC [15] or peak expiratory flow (PEF) [16] with evening versus morning administration. In each of these studies no difference was seen between once daily morning or evening administration in terms of AEs [13], including cortisol levels where assessed [15,16].

Fluticasone furoate (FF) is a novel ICS and is structurally different to fluticasone propionate (FP). FF has an ester derived from 2-furoic acid at the C-17α position that replaces the simpler propionate ester [17]. This feature of FF confers both greater affinity for and longer retention in respiratory tissues than FP [18]. FF remains active 24 hours after administration; therefore it is in development for use as a once-daily inhaled treatment for asthma. Data from an early phase clinical study demonstrated that the duration of action of FF extends beyond 24 hours and is therefore longer than that of FP, making FF potentially suitable for consideration of once-daily administration [19]. The program of phase II dose selection studies evaluating FF in asthma is now complete; findings from several of these trials have shown that FF has a favourable efficacy and safety profile when administered as a once-daily treatment for asthma [20-22].

This phase II study was designed to compare the efficacy and safety of FF 200 mcg and 400 mcg administered once daily in the morning or in the evening, with FF 200 mcg twice daily (morning and evening) in patients ≥ 12 years with persistent asthma who remained symptomatic despite low-dose ICS therapy.

## Methods

### Study design

This was a phase IIa, randomized, double-blind, parallel-group, placebo-controlled study conducted at 70 investigative sites in 16 countries around the world (clinicaltrials.gov study number NCT00398645; GSK study number FFA106783). The study was conducted between November 2006 and August 2007 and comprised a 2-week pre-treatment screening period (Day -14 to Day 0) for evaluation of eligibility and asthma status, an 8-week double-blind treatment phase, and telephonic follow-up contact 1 week after completing the study medication. During the double-blind treatment phase, patients were required to attend 5 on-treatment morning clinic visits (weeks 1, 2, 4, 6, and 8) and 3 on-treatment evening clinic visits (weeks 2, 4, and 8). Patients were issued with electronic daily diaries (eDiary; Asthma Monitor plus [AM 2+], Jaeger, Hoechberg, Germany), which were used to enter information including morning and evening PEF (measured using the AM 2+ device), daytime and night-time asthma symptom score, daytime and night-time use of salbutamol rescue medication. These data were then used to establish eligibility during the screening period and to establish a baseline from which to determine symptomatic worsening of asthma during the double-blind treatment period. Patients were also asked to record in their eDiary their use of non-study issued maintenance ICS during the screening period and their use of blinded study medication to assess compliance with study medication. eDiary data for PEF, rescue medication and symptom score were not data based or analyzed and the information collected was used by the study investigator for safety purposes only. All patients were assessed and treated on an out-patient basis.

The study was conducted in accordance with the Declaration of Helsinki and Good Clinical Practice guidelines and was approved by local ethics committees and institutional review boards as appropriate. All patients provided written informed consent prior to participating in the study.

### Patients

Male and female patients aged ≥ 12 years with a documented history of asthma as defined by the National Institute of Health [2,23] were eligible for study entry. Other inclusion criteria were baseline morning forced expiratory volume in one second (FEV_1_) 50-80% of the predicted normal value, and reversibility of baseline FEV_1 _(≥ 12% and ≥ 200 ml) in response to inhaled salbutamol. Study participants had to be able to replace their current short-acting beta_2 _agonist (SABA) therapy with salbutamol inhalation aerosol during the screening period, and to be able to withhold all inhaled short-acting beta sympathomimetic bronchodilators for 6 hours before each study visit. They also had to have been taking ICS for ≥ 3 months before screening, with a stable daily dose for 4 weeks before screening. The maximum daily ICS dose was FP 200 mcg or equivalent.

Patients were excluded if they had a history of life-threatening asthma, a respiratory infection within 4 weeks of screening, an asthma exacerbation within 4 weeks of screening, or that required oral corticosteroids within 3 months or hospitalization within 6 months of screening, clinically significant uncontrolled disease, oropharyngeal candidiasis, or a recent (1 year) or heavy (> 10 pack years) smoking history. Female patients of childbearing potential who were not using an acceptable method of contraception and patients with severe milk protein allergy or an adverse drug reaction to any beta_2 _agonist, sympathomimetic drug or intranasal, inhaled, or systemic corticosteroid were also excluded.

After the 2-week screening period, patients were randomized if morning pre-dose FEV_1 _was 50-80% of the predicted normal value and within ± 15% of the pre-bronchodilator FEV_1_, and if they continued to have symptoms requiring salbutamol use or a 24-hour asthma symptom score of ≥ 1 on at least 4 of the last 7 days of screening. Patients were excluded if during screening they had changes to their asthma medication, a lower or upper respiratory tract infection, asthma exacerbation, oral candidiasis, or were non-compliant with the eDiary.

### Study treatment

Patients who successfully completed the screening period were randomized to one of six treatments (ratio, 1:1:1:1:1:1) administered via a Diskus^®^/Accuhaler^® ^for 8 weeks: FF 200 mcg or 400 mcg once daily in the morning, FF 200 mcg or 400 mcg once daily in the evening, FF 200 mcg twice daily, or placebo (twice daily). Patients had stopped their usual ICS therapy one day prior to randomization. Patients who were randomized to once-daily treatment received a matching placebo Diskus^®^/Accuhaler^®^. Patients were instructed to administer one inhalation from one inhaler in the morning and one inhalation from the other inhaler in the evening, approximately 12 hours apart. Use of the salbutamol Diskus^®^/Accuhaler^® ^device or nebulized salbutamol (excluding for reversibility testing during screening) was not allowed during the study. The use of a salbutamol metered-dose inhaler was, however, permitted for symptom relief.

Patients, investigators and study personnel were all blinded to study treatment. The central randomization schedule was generated by the sponsor using a validated computerized system (RandAll). Patients were randomized using Registration and Medication Ordering System (RAMOS), an automated, interactive telephone based system, which was used by the investigator or designee to register and randomize the patient and receive medication assignment information.

Patients were observed by appropriately trained site personnel during each clinic visit to ensure that they were able to administer the study drug correctly. The eDiary was used to question patients on their compliance with study medication each morning and evening; patients who were not compliant were counselled on the appropriate way to administer the study drug.

The following anti-asthma medications were not allowed ≤ 2 weeks before screening or during the study: combination therapy comprising an inhaled beta_2 _agonist and ICS, slow-release bronchodilators, anticholinergics, long-acting beta_2 _agonists (LABA), ketotifen, nedocromil sodium, sodium cromoglycate, and oral LABA. Other drugs prohibited before screening included oral SABA (within 24 hours), anti-leukotrienes or potent CYP3A4 inhibitors (within 4 weeks), and systemic, oral, parenteral, or depot corticosteroids, or anti-IgE therapy (within 3 months). Immunotherapy was permitted if initiated before screening and used at a stable dose for the treatment of allergies. Drug therapies for other medical conditions, with the exception of systemic corticosteroids, were permitted throughout the study provided the dose remained constant and their use was not expected to affect the patient's lung function or asthma status.

### Efficacy assessment

The primary, single, efficacy endpoint was the mean change from baseline at week 8 in the pre-dose (morning or evening [depending on regimen], pre-rescue bronchodilator) FEV_1 _(measured using a MasterScope CT spirometer [Viasys, Hoechberg, Germany]). This was recorded electronically at the morning clinic visits between 6 am and 11 am or at the evening clinic visits between 6 pm and 11 pm using flow-volume curves generated from calibrated spirometers and according to ATS/ERS guidelines [24]. Patients were required to withhold their salbutamol therapy for ≥ 6 hours before each clinic visit. Treatment compliance was derived from the number of positive answers in the patient's eDiary, which were divided by the number of non-missing answers and expressed as a percentage for both morning and evening doses.

### Safety evaluation

The following safety endpoints were evaluated: incidence of adverse events (AEs) and serious AEs (SAEs), vital signs, hematology, clinical chemistry, and urinalysis parameters, oropharyngeal examinations, and withdrawals due to worsening asthma. AEs/SAEs were coded using the Medical Dictionary for Regulatory Activities. Twenty-four hour urinary-free cortisol excretion was also measured at baseline (week 0) and at the end of the double-blind treatment period (week 8) to assess hypothalamic-pituitary-adrenal axis (HPA) function. A central laboratory was used for all cortisol measurements.

### Statistical analysis

Assuming a common standard deviation of 450 ml, a sample size of 648 patients (108 per group) was required to provide 90% power to detect a treatment difference of 200 ml in pre-dose FEV_1 _between FF and placebo at the (two-sided) 5% significance level. The study was not powered to formally assess differences between once-daily treatment and twice-daily treatment or differences between morning treatment and evening treatment, therefore statistical comparisons of all FF treatment groups were only against placebo. However it was pre-specified in the study protocol that provided the FF treatment groups demonstrated a statistically significant difference relative to placebo, the relative effects of once-daily and twice-daily dosing and of morning and evening dosing would be evaluated by assessing the degree of overlap between the 95% confidence intervals relating to the treatment differences with placebo. If the point estimate of the treatment/placebo difference for any given FF regimen lay within the 95% confidence interval for another FF regimen, the treatment effect estimates would be within 0.12 L of each other.

The intent-to-treat (ITT) population, which included all randomized patients who received at least one dose of study medication, was the primary population for all efficacy and safety (excluding urinary cortisol) analyses. The per protocol (PP) population (all subjects in the ITT population who did not have any full protocol deviations) was used for confirmatory analysis of the primary endpoint.

Analysis of the primary efficacy endpoint was conducted using an analysis of covariance (ANCOVA) model with effects due to baseline pre-dose FEV_1_, country, sex, age, and treatment group. Any patient with a missing FEV_1 _measurement at week 8 was included in the analysis of the primary endpoint by imputation using the preceding non-missing FEV_1 _value (last observation carried forward). The analysis was performed separately for morning and evening time-points, with the placebo and twice-daily regimens used in both cases. Estimated treatment differences for pair-wise comparisons against placebo were presented together with 95% confidence intervals for the difference and p values.

The patient population for urinary cortisol (UC) analyses comprised all patients whose urine samples were not considered to have confounding factors that would affect the interpretation of the results. The 24-hour UC excretion was log-transformed and analyzed using an ANCOVA model with effects due to baseline, country, sex, age, and treatment group.

## Results

### Patients

**Figure 1:**
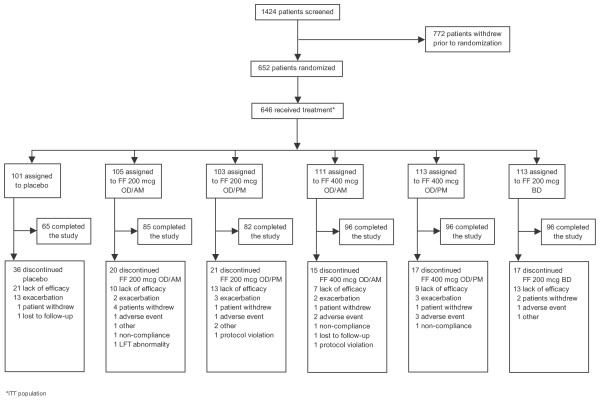
**Study design (CONSORT)**.

A total of 1424 patients were screened with 652 patients randomized into the study. A total of 646 randomized patients received at least one dose of study drug and comprised the ITT population. Of these patients, 101 were assigned to placebo, 105 and 103, respectively, received FF 200 mcg once daily in the morning or evening, 111 and 113, respectively, received FF 400 mcg once daily in the morning or evening, and 113 were allocated to FF 200 mcg twice daily. The study was completed by 65 (64%) placebo-treated patients and 455 (83%) FF-treated patients. A total of 597 patients had no full protocol deviations and therefore comprised the PP population. Reasons for patient withdrawal at the screening and randomization stages are summarized in Figure 1.

All treatment groups were comparable and well matched with respect to baseline demographic and disease characteristics (Table 1). Mean age of the study population overall was 45.1 years, with a higher proportion of females. More than half of the patients (57%) had at least a 10-year history of asthma and lung function was similar across all 6 treatment groups.

**Table 1:** Baseline characteristics (ITT population)

Demographic Characteristics	Placebo (*n *= 101)	FF dose				
		
		200 mcg OD/AM (*n *= 105)	200 mcg OD/PM (*n *= 103)	400 mcg OD/AM (*n *= 111)	400 mcg OD/PM (*n *= 113)	200 mcg BD (*n *= 113)
Female gender, n (%)	62 (61)	62 (59)	74 (72)	73 (66)	70 (62)	78 (69)

Age (years), mean	44.4	45.0	43.7	46.9	45.0	45.6

Race, n (%)^†^						

White	60 (60)	68 (65)	67 (66)	74 (67)	75 (68)	76 (67)

Asian	16 (16)	14 (13)	15 (15)	16 (15)	15 (14)	17 (15)

Other	24 (24)	22 (22)	20 (20)	20 (18)	21 (19)	20 (18)

Asthma history, n (%)						

< 1 year	3 (3)	1 (< 1)	3 (3)	2 (2)*	3 (3)	3 (3)*

≥ 1 to < 5 years	16 (16)	12 (11)	19 (18)	14 (13)	19 (17)	17 (15)

≥ 5 to < 10 years	24 (24)	26 (25)	26 (25)	30 (27)	24 (21)	35 (31)

≥ 10 years	58 (57)	66 (63)	55 (53)	65 (59)	67 (59)	58 (51)

Lung function, mean						

Pre-bronchodilator FEV_1_, litres	1.966	1.969	1.986	1.931	1.995	1.976

% predicted FEV_1_, (%)	66.37	66.52	68.24	67.23	67.69	68.14

% reversibility FEV_1_, (%)	30.16	29.25	29.29	27.94	30.90	26.32

*1 patient < 6 months

^†^Data on race unavailable for 6 patients

AM = morning dosing; BD = twice daily; FEV_1 _= forced expiratory volume in one second; OD = once daily; PM = evening dosing

Treatment compliance during the active treatment phase was similar across all treatment groups. Mean overall compliance ranged from 97.4-99.1% and from 96.6-98.4% for the morning and evening dosing regimens, respectively. Mean exposure to study drug was 43.1 days in the placebo group and ranged from 50.3-52.2 days in the FF groups; exposure was highest in the FF 400 mcg once-daily morning group and lowest in the FF 200 mcg once-daily evening group.

### Efficacy

**Figure 2:**
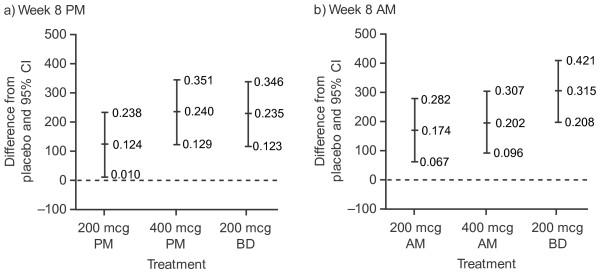
**Adjusted treatment difference in pre-dose FEV_1 _(ml) (last observation carried forward; ITT population)**.

There were statistically significant improvements in pre-dose FEV_1 _for each FF treatment arm compared with placebo (Figure 2, Table 2). FF 400 mcg once daily in the evening resulted in similar placebo-adjusted improvements in evening pre-dose FEV_1 _at week 8 compared with FF 200 mcg twice daily (240 ml vs. 235 ml). FF 200 mcg twice daily resulted in greater improvements in placebo-adjusted morning pre-dose FEV_1 _than 400 mcg once daily in the morning at week 8 (315 ml vs. 202 ml).

**Table 2:** Change from baseline in trough FEV_1 _(litres) (last observation carried forward) at week 8, by evening (PM) vs. morning (AM) FEV_1 _and treatment group (ITT population)

**Week 8 PM FEV**_**1**_				
	**Placebo** **(*n *= 101)**	**FF dose**		
		
		**200 mcg OD/PM** **(*n *= 103)**	**400 mcg OD/PM** **(*n *= 113)**	**200 mcg** **BD** **(*n *= 113)**

Trough FEV_1 _(n)	77	92	103	100

LS mean (SE)	2.198 (0.0458)	2.322 (0.0437)	2.438 (0.0398)	2.432 (0.0411)

LS mean change (SE)	0.084 (0.0458)	0.208 (0.0437)	0.324 (0.0398)	0.319 (0.0411)

Difference from placebo				

Difference		0.124	0.240	0.235

95% CI		0.010, 0.238	0.129, 0.351	0.123, 0.346

p value		0.033	< 0.001	< 0.001

**Week 8 AM FEV**_**1**_				

	**Placebo** **(*n *= 101)**	**FF dose**		
		
		**200 mcg OD/AM** **(*n *= 105)**	**400 mcg OD/AM** **(*n *= 111)**	**200 mcg** **BD** **(*n *= 113)**

Trough FEV_1 _(n)	85	100	106	102

LS mean (SE)	2.029 (0.0434)	2.203 (0.0389)	2.230 (0.0397)	2.344 (0.0400)

LS mean change (SE)	0.053 (0.0434)	0.228 (0.0389)	0.255 (0.0397)	0.368 (0.040)

Difference from placebo				

Difference		0.174	0.202	0.315

95% CI		0.067, 0.282	0.096, 0.307	0.208, 0.421

p value		0.002	< 0.001	< 0.001

BD = twice daily; CI = confidence interval; FEV_1 _= forced expiratory volume in one second; FF = fluticasone furoate; LS = least square; OD = once daily; SE = standard error

**Figure 3:**
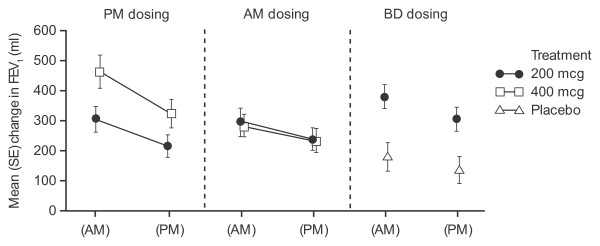
**Comparison of AM and PM FEV_1 _at week 8 following once-daily dosing in the evening or morning, or twice-daily dosing (ITT population)**.

A ≥ 200 ml increase in placebo-adjusted pre-dose FEV_1 _was observed for FF 400 mcg administered once daily in the morning or evening and for FF 200 mcg twice daily but not for either of the FF 200 mcg once-daily dose groups. Nevertheless, increase from baseline was ≥ 200 ml with both FF 200 mcg once-daily dose groups. The week 8 AM and PM FEV_1 _responses for each treatment are shown in Figure 3.

Because all FF treatments resulted in a significant difference from placebo the relative effects of once-daily and twice-daily dosing and of morning and evening dosing were evaluated by assessing the degree of overlap between the 95% confidence intervals relating to the treatment differences with placebo. The point estimate for the difference from placebo for morning pre-dose FEV_1 _for 400 mcg dosed once daily in the morning was not within the 95% confidence interval for the placebo adjusted change from baseline in morning FEV_1_seen with FF 200 mcg dosed twice-daily (202 ml point estimate for FF 400 mcg OD vs. 208 ml lower 95% confidence interval for FF 200 mcg BD). In contrast the point estimate for the placebo adjusted change from baseline in evening FEV_1 _for 400 mcg once daily in the evening was within the 95% confidence interval for the placebo adjusted change from baseline in evening FEV_1 _seen with FF 200 mcg twice daily, indicating that the efficacy of FF 400 mcg once daily in the evening is similar to that of FF 200 mcg twice daily.

Results for the PP population were consistent with those of the ITT population although the relative treatment effect of all FF treatment groups was generally lower. The effect of FF 200 mcg once daily dosed in the evening on pre-dose (evening) FEV_1 _was not significantly different from placebo (p = 0.264).

### Safety

The proportion of patients who reported any AE during the treatment period was 28% in the placebo group and 31-39% in the FF treatment groups. The most frequently reported AEs during treatment were headache (6-9%), nasopharyngitis (3-8%), bronchitis (0-4%), pharyngolaryngeal pain (< 1-3%), and upper respiratory tract infection (< 1-3%) (Table 3). The incidence and type of AEs were generally similar to placebo and the frequency of AEs did not appear to be related to the dose of FF.

**Table 3:** Most common on-treatment AEs (≥ 3% incidence in any treatment group) (ITT population)

	Placebo (*n *= 101)	FF dose				
		
		200 mcg OD/AM (*n *= 105)	200 mcg OD/PM (*n *= 103)	400 mcg OD/AM (*n *= 111)	400 mcg OD/PM (*n *= 113)	200 mcg BD (*n *= 113)
**Any AE, n (%)**	28 (28)	36 (34)	32 (31)	43 (39)	35 (31)	38 (34)

Headache	6 (6)	8 (8)	7 (7)	10 (9)	7 (6)	9 (8)

Nasopharyngitis	4 (4)	8 (8)	8 (8)	3 (3)	7 (6)	6 (5)

Bronchitis	2 (2)	1 (< 1)	3 (3)	4 (4)	4 (4)	0

Pharyngolaryngeal pain	1 (< 1)	2 (2)	3 (3)	2 (2)	1 (< 1)	3 (3)

Upper respiratory tract infection	2 (2)	3 (3)	2 (2)	2 (2)	1 (< 1)	1 (< 1)

Dysphonia	0	1 (< 1)	1 (< 1)	1 (< 1)	2 (2)	3 (3)

Rhinitis	0	4 (4)	1 (< 1)	0	1 (< 1)	2 (2)

Rhinitis allergic	1 (< 1)	2 (2)	3 (3)	0	0	1 (< 1)

Dizziness	0	3 (3)	0	2 (2)	1 (< 1)	0

Influenza	2 (2)	0	1 (< 1)	3 (3)	0	0

Pharyngitis	4 (4)	2 (2)	0	0	0	0

Respiratory tract infection	0	1 (< 1)	0	3 (3)	1 (< 1)	0

AE = adverse event; AM = morning dosing; BD = twice daily; OD = once daily; PM = evening dosing

A total of four SAEs were reported. Two of these occurred on treatment and included one case of angioedema in the FF 200 mcg once-daily evening group and one case of atrial fibrillation in the FF 400 mcg once-daily morning group. Two other SAEs were reported post-treatment and included one case of spontaneous abortion and one cerebrovascular accident (ischemic lesion) in two separate patients both in the FF 200 mcg twice-daily group. All 4 patients required hospitalization for their SAEs; however, only the case of angioedema was considered to be possibly related to the study drug. The patient was subsequently withdrawn from the study and treated with intravenous hydrocortisone after which her condition improved with no sequelae.

A total of 11 patients reported 13 AEs that resulted in study withdrawal: 3 patients in the FF 200 mcg once-daily morning group, 1 in the FF 200 mcg once-daily evening group, 3 in the FF 400 mcg once-daily morning group, 3 in the FF 400 mcg once-daily evening group and 1 in the FF 200 mcg twice-daily group. Two of these events (angioedema and cerebrovascular accident, already described) met the criteria for an SAE. Nine of the AEs were considered by the investigator to possibly be related to the study drug and included swelling of the eyelids and lips and increased blood pressure (FF 200 mcg once-daily morning), acute localized Quincke's oedema of the eyes (FF 200 mcg once-daily evening), headache and elevated gamma glutamyl transferase levels (FF 400 mcg once-daily morning) and oedema, facial rash, and hypersensitivity reaction (FF 400 mcg once-daily evening). No deaths were reported during the study. There were no safety concerns related to vital signs, or laboratory safety tests. No treatment-related changes were apparent. The incidence of oral candidiasis was low in the FF treatment groups (0-4% vs. < 1% for placebo) as was the incidence of asthma exacerbations (< 1-4% vs. 14% for placebo).

**Figure 4:**
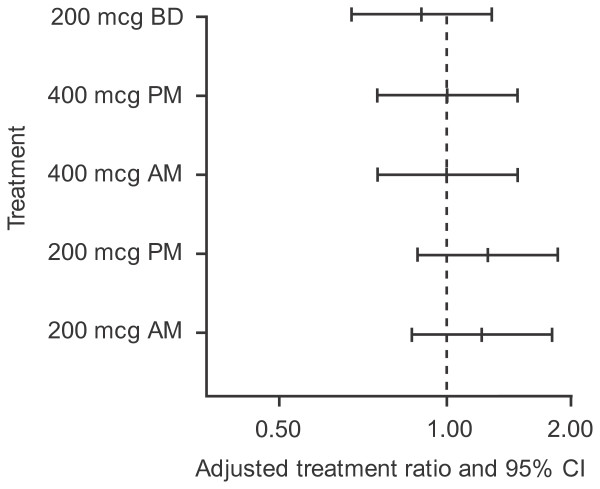
**Adjusted treatment ratio for 24-hour urinary cortisol excretion for each FF treatment group vs. placebo (UC population). Error bars are 95% confidence intervals**.

24-hour UC excretion at week 8 was similar across treatment groups with adjusted geometric means of 48.24 nmol/24 hour for placebo and 43.41-57.23 nmol/24 hour for the FF treatment groups. The adjusted ratios to baseline were 0.87 for placebo and 0.78-1.03 for the FF treatment groups and the adjusted ratios to placebo for the FF treatment groups ranged from 0.90-1.19 (Figure 4).

## Discussion

This study was designed to assess the efficacy and safety of FF 200 mcg or 400 mcg administered once daily in the morning or evening and FF 200 mcg administered twice daily in the morning and evening in patients with mild to moderate asthma who were still symptomatic despite treatment with low-dose ICS therapy. FF 400 mcg administered once-daily in the morning or evening and FF 200 mcg administered twice daily (approximately 12 hours apart) each resulted in clinically and statistically significant improvements in pre-dose FEV_1_, as defined by an increase of ≥ 200 ml, compared with placebo, after 8 weeks of dosing. Furthermore, FF 400 mcg once-daily dosing in the evening resulted in a similar improvement in pre-dose FEV_1 _to FF 200 mcg twice daily. However, although FF 400 mcg once daily in the morning resulted in a statistically and clinically significant improvement in FEV_1 _compared with placebo, the improvement was slightly less than that seen with FF 200 mcg twice daily. Both FF 200 mcg once-daily dosing regimens did not increase the trough FEV_1 _by ≥ 200 ml, vs. placebo after 8 weeks of dosing, though a ≥ 200 ml change from baseline was observed with both regimens.

Although not powered to show equivalence between the doses or scheduling of FF treatment, the results of this study indicate that FF dosed at 400 mcg once-daily in the evening resulted in similar efficacy to FF 200 mcg dosed twice-daily. This was not the case for FF 400 mcg dosed once-daily in the morning, where the point estimate for the placebo adjusted change from baseline in morning FEV_1 _was lower than the lower boundary for the 95% confidence interval for the placebo adjusted change from baseline in morning FEV_1 _seen with FF 200 mcg dosed twice-daily.

The findings from our trial are, in part, similar to results from earlier double-blind studies evaluating once-daily therapy with other ICS for asthma. In a 12-week study, mometasone furoate 200 mcg administered once daily in the evening was more effective than mometasone furoate 200 mcg administered once daily in the morning in terms of change in FEV_1 _and forced vital capacity (FVC) [15]. Similar improvements in lung function with evening compared with morning FF 400 mcg dosing were reported in our study. However, in the once-daily mometasone furoate 200 mcg study, the change from baseline in FEV_1 _and FVC with morning dosing was not significantly different from placebo [15]. In contrast, our study demonstrated significant improvements from baseline in FEV_1 _vs. placebo with both morning and evening FF 200 mcg and FF 400 mcg once-daily dosing. In a second study, budesonide 400 mcg administered once daily in the morning or evening was as effective as budesonide 200 mcg administered twice daily for 12 weeks. Mean differences between placebo and active treatments at weeks 11 and 12 were higher for the 400 mcg once-daily evening regimen compared with the morning regimen in terms of both morning and evening PEF [13]. Similarly, ciclesonide 200 mcg once daily dosed in the evening compared with morning dosing resulted in greater improvements in morning (12-hour treatment) and evening (24-hour treatment) PEF relative to baseline at 8 weeks [16]. However, more recent studies have reported that twice daily dosing with 80 mcg ciclesonide is generally more efficacious than once daily dosing with 160 mcg ciclesonide (in the morning) in improving and maintaining disease control, over treatment periods of 12-16 weeks [25,26].

The duration of action of the majority of currently available ICS necessitates twice-daily administration. Only one ICS (mometasone furoate) is currently recommended for once-daily dosing in both the US and Europe but only for use in patients with non-severe asthma [2,27]. Thus, at present the potential advantages of once daily dosing are not available for patients of all asthma severities. Non adherence to treatment is an important issue in asthma management, particularly in patients on long-term ICS and other controller medications [2,28]. In a retrospective analysis of treatment adherence and markers of asthma control in patients with mild asthma, adherence (measured by percentage days covered) to twice-daily ICS was just 14.5% [29]. Other studies have shown that regular use of ICS is associated with a reduction in hospitalization or emergency room visits due to asthma [30,31]. Therefore, lack of adherence and sub-optimal use of ICS therapy highlights the potential benefits of a convenient once-daily ICS that provides effective asthma control over a 24-hour period across a broad spectrum of asthma patients.

In the current study, FF was generally well tolerated and the incidence of AEs with FF was low and generally similar to placebo; however, three cases of oedema leading to withdrawal were reported with FF (one in each of three different treatment groups). This study was not powered for safety and these events will be assessed as we compile more information during the drug development programme. Data from other phase II studies evaluating FF have reported similar safety findings with daily doses of up to 600 mcg [20-22] as those reported here, save for no withdrawals due to oedema. In these other studies, headache, upper respiratory tract infection, oropharyngeal pain, nasopharyngitis, and sinusitis were the most frequently reported AEs and occurred with a similar incidence to placebo. The incidence of oral candidiasis was typically low at < 1-4% [20-22]. At the moderate and high doses required to treat some patients with asthma, ICS therapy can lead to suppression of the HPA axis [32] and it has been suggested that this effect may be accentuated by once-daily dosing in the evening [33,34]. However, safety data from our study suggest that FF 200 or 400 mcg administered once daily in the evening has a minimal effect on UC levels; 24-hour urinary excretion ratios were similar to placebo for all FF treatment groups and between morning and evening dosing and once-daily and twice-daily dosing. These data are consistent with previously published studies. One study showed no difference in placebo-adjusted 24-hour UC results for budesonide 400 mcg administered once daily in the morning compared with evening [35]. In another study, there was no significant difference in morning serum cortisol levels, between patients receiving mometasone furoate dosed once-daily in the evening (1 inhalation of 400 mcg or 2 inhalations of 200 mcg) and those receiving placebo, also in the evening [36]. In addition, phase II studies with FF have demonstrated similar UC levels for different doses of FF given once daily in the evening compared with placebo [20-22].

The design of the current study utilized pre-dose FEV_1 _as the primary endpoint because it is a reliable index of 24-hour duration of action and the study was adequately powered to demonstrate treatment differences on this basis. The inclusion of a placebo control group enabled the magnitude of effect of FF on efficacy and safety parameters to be established and also allowed variability in 24-hour UC excretion values to be determined for comparison against the active treatment groups. An 8-week treatment period was selected as an appropriate duration to observe changes in pulmonary function from baseline and to permit assessment of differences between treatments. A limitation of the study was that it was not powered to demonstrate non-inferiority of the once-daily treatment groups relative to the twice-daily treatment group or of the morning dosing regimens relative to the evening dosing regimens; therefore statistical comparisons of all FF treatment groups were only compared with placebo. Furthermore, as dosing frequency can impact the efficacy of ICS therapy, particularly in patients with uncontrolled or severe asthma [37], conclusions drawn from this population of patients with mild to moderate asthma preclude generalizations of efficacy and safety to patients with higher levels of uncontrolled asthma and symptom severity.

## Conclusions

Once-daily treatment with FF 400 mcg dosed in the evening or morning showed clinically and statistically significant improvements in pre-dose FEV_1 _(≥ 200 ml). FF at both doses and all regimens was generally well tolerated. FF 400 mcg administered once daily in the evening had similar efficacy to FF 200 mcg administered twice daily. These findings suggest that FF is an effective and well tolerated once-daily ICS, although additional confirmatory studies are required.

## Competing interests

AW has served as consultant to Almirall, AstraZeneca, Chiesi, GlaxoSmithKline, Merck Sharpe and Dohme, Novartis and Schering Plough; and has received research grants and travel expenses for attendance at ATS and ERS meetings from GlaxoSmithKline.

ERB has served as a consultant to GlaxoSmithKline; and has performed clinical trials for GlaxoSmithKline, which have been administered by his employer Wake Forest University Health Sciences.

EDB has served as a consultant to and received lecture fees from GlaxoSmithKline; and his institution has received remuneration for participation in clinical trials sponsored by GlaxoSmithKline.

WWB has served as a consultant to AstraZeneca, Boehringer Ingelheim, Novartis and TEVA; served on advisory boards for Altair, Amgen, Centocor, GlaxoSmithKline, Johnson & Johnson, Merck Sharpe and Dohme and Pfizer; received lecture fees from Merck Sharpe and Dohme; and received research funding from AstraZeneca, Ception, GlaxoSmithKline, MedImmune and Novartis.

JL has served as a consultant to and received lecture fees from AstraZeneca, GlaxoSmithKline, Merck Sharpe and Dohme, Novartis and UCB Pharma; has been partly covered by some of these companies to attend previous scientific meetings including the ERS and the AAAAI; and has participated in clinical research studies sponsored by AstraZeneca, GlaxoSmithKline, Merck Sharpe and Dohme, and Novartis. JL is also editor of Respiratory Research and recused himself fully from the editorial process of this manuscript.

NGS, RF, LJ and BH are employees of and hold stock/shares in GlaxoSmithKline.

## Authors' contributions

All listed authors meet the criteria for authorship set forth by the International Committee for Medical Journal Editors. AW, EDB, WB, JL, LJ, BH and ERB participated in concept approval, reviewed data and edited the manuscript. NGS was the clinical investigation leader, contributed to running the study and edited the manuscript. RF conducted statistical analysis of the data and edited the manuscript. All authors were involved in the design and concept of the study, had full access to and interpreted the data and contributed to the development of the manuscript. All authors can vouch for the veracity and completeness of the data and data analysis. All authors read and approved the final manuscript.
